# Mycophenolic acid, the active form of mycophenolate mofetil, interferes with IRF7 nuclear translocation and type I IFN production by plasmacytoid dendritic cells

**DOI:** 10.1186/s13075-020-02356-z

**Published:** 2020-11-09

**Authors:** Minoru Shigesaka, Tomoki Ito, Muneo Inaba, Kai Imai, Hideki Yamanaka, Yoshiko Azuma, Akihiro Tanaka, Hideki Amuro, Tohru Nishizawa, Yonsu Son, Atsushi Satake, Yoshio Ozaki, Shosaku Nomura

**Affiliations:** First Department of Internal Medicine, Kansai Medical University, 2-5-1 Shin-machi, Hirakata City, Osaka 573-1010 Japan

**Keywords:** SLE, Plasmacytoid DCs, Myeloid DCs, Type I IFN, MPA, IRF7

## Abstract

**Background:**

Both humoral and cellular immune mechanisms are involved in the onset and progression of autoimmune responses in systemic lupus erythematosus (SLE). Plasmacytoid dendritic cells (pDCs) play a central role in the pathogenesis of SLE via the dysregulation of type I interferon (IFN) production; these cells act together with activated myeloid DCs (mDCs) to amplify the vicious pathogenic spiral of autoimmune disorders. Therefore, control of aberrant DC activation in SLE may provide an alternative treatment strategy against this disease. Mycophenolate mofetil (MMF), which has been used to treat lupus nephritis, specifically blocks the proliferation of B and T lymphocytes via inhibition of inosine-5-monophosphate dehydrogenase. Here, we focus on the effects of MMF in targeting DC functions, especially the IFN response of pDCs.

**Methods:**

We isolated human blood pDCs and mDCs by flow cytometry and examined the effect of mycophenolic acid (MPA), which is a metabolic product of MMF, on the toll-like receptor (TLR) ligand response of DC subsets. Additionally, we cultured pDCs with serum from SLE patients in the presence or absence of MPA and then examined the inhibitory function of MPA on SLE serum-induced IFN-α production.

**Results:**

We found that treatment with 1−10 μM of MPA (covering the clinical trough plasma concentration range) dose-dependently downregulated the expression of CD80 and CD86 on mDCs (but not pDCs) without inducing apoptosis, in response to R848 or CpG-ODN, respectively. Notably, in pDCs, MPA significantly suppressed IFN-α production with IRF7 nuclear translocation and repressed the AKT activity. In addition, MPA inhibited IL-12 production with STAT4 expression in mDCs. We further identified that MPA had an inhibitory effect on SLE serum-induced IFN-α production by pDCs.

**Conclusions:**

Our data suggest that MPA can interrupt the vicious pathogenic spiral of autoimmune disorders by regulating the function of DC subsets. This work unveiled a novel mechanism for the therapeutic ability of MMF against SLE.

## Background

Mycophenolate mofetil (MMF), which has been used to treat lupus nephritis as well as in solid organ transplantation, is an uncompetitive and reversible inhibitor of inosine monophosphate dehydrogenase (IMPDH) [1, 2]. In the body, MMF is naturally hydrolyzed into mycophenolic acid (MPA), which is the active form of MMF. MMF/MPA inhibits the de novo pathway of guanosine nucleotide synthesis without incorporation into DNA [3]. Because T and B lymphocytes depend on the de novo synthesis of purines for their proliferation, MMF/MPA has potent cytostatic effects on lymphocytes [2]. MMF has been shown to prevent the occurrence of acute rejection in rat models of kidney and heart allotransplantation [4]. MMF also decreases antibody production in mice [5]. Thus, MMF functions as a versatile immune modulator because of its broad range of actions. The biological mechanisms underlying the therapeutic effects of MMF/MPA against rejection after organ transplantation are known to be mainly mediated by the inhibition of T and B lymphocytes, but the mechanisms by which MMF/MPA improves lupus nephritis are still largely unclear.

Dendritic cells (DCs) orchestrate both innate and adaptive immunity through cytokine-mediated and cell−cell contact mechanisms. In humans, DCs consist of two major subsets: myeloid DCs (mDCs) and plasmacytoid DCs (pDCs). They play distinct roles in innate and adaptive immunities through their capability to express specialized cytokines and molecules [6–8]. Although they account for only a small fraction of cells, pDCs represent a major source of type I interferon (IFN) in the peripheral blood and lymphoid tissues [9]. Thus, they play a central role in the innate antiviral immune response via their ability to produce vast amounts of type I IFN. This function is based on their selective expression of toll-like receptor (TLR)7 and TLR9, which respectively sense viral RNA and DNA within early endosomes [10]. Additionally, pDCs constitutively express high levels of IFN regulatory factor 7 (IRF7) [11], and type I IFN production requires IRF7 to be phosphorylated and translocated into the nucleus through rapid interaction with MyD88 and IRF7 [12–14].

Notably, a series of studies have indicated that pDCs also play a pathogenic role in the development of autoimmune diseases, such as systemic lupus erythematosus (SLE) or psoriasis, through their specialized function in type I IFN production [15–17]. SLE is a heterogeneous disease in which excessive inflammation, autoantibodies, and complement activation lead to multisystem tissue damage. The vicious pathogenic spiral responsible for the autoimmune process in SLE, likely mediated by the sustained secretion of type I IFN [15], is thought to occur as follows: pDC-derived type I IFN rapidly triggers the differentiation and activation of myeloid DCs, which in turn elicits the differentiation of autoreactive CD4^+^ T cells, CD8^+^ T cells, and B cells as antigen-presenting cells through processing self-components. These autoreactive effectors induce tissue damage (thus producing DNA/RNA fragments) and produce auto-anti-nuclear antibody. The resultant immune complexes, such as anti-DNA antibody−self-DNA, further activate pDCs through TLRs in a sustained manner to produce type I IFN, which in turn amplifies the vicious autoimmune spiral. Consequently, pDCs and type I IFN represent specific cellular and molecular targets, respectively, in therapeutic strategies against autoimmune diseases such as SLE.

Although there is evidence indicating that MMF/MPA has immunomodulatory effects on mDCs [18–20], there are no reports showing its effects on pDCs. Therefore, the present work focused on examining the effects of MMF/MPA on the blood DC subsets, especially pDCs.

Here, we show a novel function of MMF/MPA, i.e., the ability to inhibit IFN-α production by human pDCs upon cellular activation with SLE serum or CpG-ODN via inhibiting the nuclear translocation of IRF7. In addition, MPA was found to suppress mDC-derived IL-12 via inhibiting STAT4 activation. Our present results suggest that MMF/MPA can interfere with the vicious pathogenic spiral of autoimmune disorders by regulating the function of DC subsets.

## Materials and methods

### Media and reagents

RPMI-1640 supplemented with 2 mM l-glutamine, 100 U/ml penicillin, 100 ng/ml streptomycin, and heat-inactivated 10% FBS (Biosource International) was used for cell culturing throughout the experiments. For human cell stimulation, we used 5 μM CpG-ODN 2216 (Invivogen), 1 μg/ml R848 (InvivoGen), and different doses of MPA (Sigma) dissolved in methanol; as a vehicle control, methanol was diluted in parallel.

### Cell isolation and culture

Human mDCs and pDCs were isolated from peripheral blood mononuclear cells (PBMCs) from healthy adult donors, as described previously [3, 21]. CD11c^+^/BDCA4^−^/lineage^−^/CD4^+^ cells (defined as mDCs) and CD11c^−^/BDCA4^+^/lineage^−^/CD4^+^ cells (defined as pDCs) were sorted by FACS Aria® (BD Biosciences) (Suppl. Fig. 1) to reach greater than 99% purity according to re-staining with anti-BDCA1 and anti-BDCA2 antibodies. The purified mDCs and pDCs were incubated for 24 h with R848 and CpG-ODN, respectively, in flat-bottomed 96-well plates at a concentration of 2 × 10^4^ cells in a final volume of 200 μl of medium per well, in the presence of MPA or the vehicle control.

### Stimulation of lupus serum

Experiments using lupus serum to stimulate pDCs were conducted as described previously [22]. Lupus sera were obtained from five active SLE patients with low complement levels prior to steroid therapy who satisfied 2019 European League Against Rheumatism (EULAR)/American College of Rheumatology (ACR) classification criteria for SLE. All patients had anti-double-stranded DNA antibody. Written informed consent was obtained from all SLE patients. In these experiments, pDCs were stimulated with 20% lupus serum in flat-bottomed 96-well plates at a concentration of 2 × 10^4^ cells in 200 μl of medium per well.

### Analyses of cells and supernatants

Human DC subsets were stained with PE-labeled CD86 (2331) and FITC-labeled CD80 (L307.4) monoclonal antibodies (all from BD Biosciences) and then analyzed by FACScalibur® (BD Biosciences). The production of cytokines in the culture supernatants after 24 h was determined by an enzyme-linked immunosorbent assay (ELISA). The ELISA kits for detecting human IL-12p40 and TNF were purchased from R&D Systems. The ELISA kit for human IFN-α was purchased from PBL Biomedical Laboratories.

For the viability assay, cells were washed with PBS containing 2 mM EDTA, and viable cells were counted in triplicate, using trypan-blue exclusion to identify the dead cells. Viable cells were also evaluated as the annexin V-negative fraction using an Annexin V–FITC Kit (Sigma).

For intracellular staining, pDCs were stimulated with CpG-ODN 2216 for 90 min or 3 h, and the cells were immediately fixed and stained with Alexa Fluor-647 anti-pAKT (pS473; BD Biosciences) (after 90 min-culture) and Alexa Fluor-anti-human IRF-7 (G-8; sc-74472 AF488, Santa Cruz Biotechnology) (after 3-h culture) using a BD Cytofix/Cytoperm™ Fixation and Permeabilization Solution (BD Biosciences) according to the manufacturer’s instructions. Then, the cells were analyzed by flow cytometry.

### Real-time PCR

Purified DC subsets were stimulated with R848 or CpG-ODN in the presence of 10 μM MPA for 4 or 8 h. The cells were then collected for cDNA synthesis using the QuantiTect. Reverse Transcription (RT) kit (QIAGEN). The DNA resulting from each RT reaction was subjected to real-time PCR using the SYBR® Green Master Mix (QIAGEN) for the detection of CD80, CD86, IFNA1, IL12B, TNF, IRF7, and STAT4. Primers for CD80 (QT00000497), CD86 (QT00033915), IL12B (QT00000364), IRF7 (QT00210595), and STAT4 (QT00014133) were purchased from QIAGEN. Primers for IFNA1 and TNF were purchased from Bio-Rad and Sino Biological, respectively. We selected glyceraldehyde 3-phosphate dehydrogenase (GAPDH) as the reference gene. Quantification of the mRNA transcription level was determined using the comparative cycle threshold (Ct) method. All samples were examined in duplicate, and the mean Ct value was calculated by Rotor Gene Q software version 2.2.3. The fold change in gene transcription was calculated as the ratio of Δ/ΔCt in the treated samples to untreated samples.

### Fluorescence microscopy

Purified pDCs were stimulated with CpG-ODN in the presence or absence of 10 μM MPA for 3 h. The cells were then seeded on glass slides by a cytospin, mounted, and fixed using a BD Transcription Factor Buffer set (Cat. No. 562725).

Samples were labeled with Alexa Fluor-anti-human IRF-7 and 4′,6′-diamidino-2-phenylindole (DAPI). Images were acquired using a fluorescence microscope (BZ-9000, Keyence). Cells were regarded as negative when the expression level of IRF7 in the cytoplasm was higher and distinguishable from that in the nucleus, as shown by DAPI staining. The ratio of nuclear IRF7-negative cells was calculated based on 50 cells from each donor.

### Statistical analysis

Data were analyzed using paired *t* tests. A value of *p* < 0.05 was considered statistically significant. Data analysis was carried out using Prism (GraphPad Software).

## Results

### Clinical concentrations of MPA do not affect the survival of human DC subsets

**Fig. 1:**
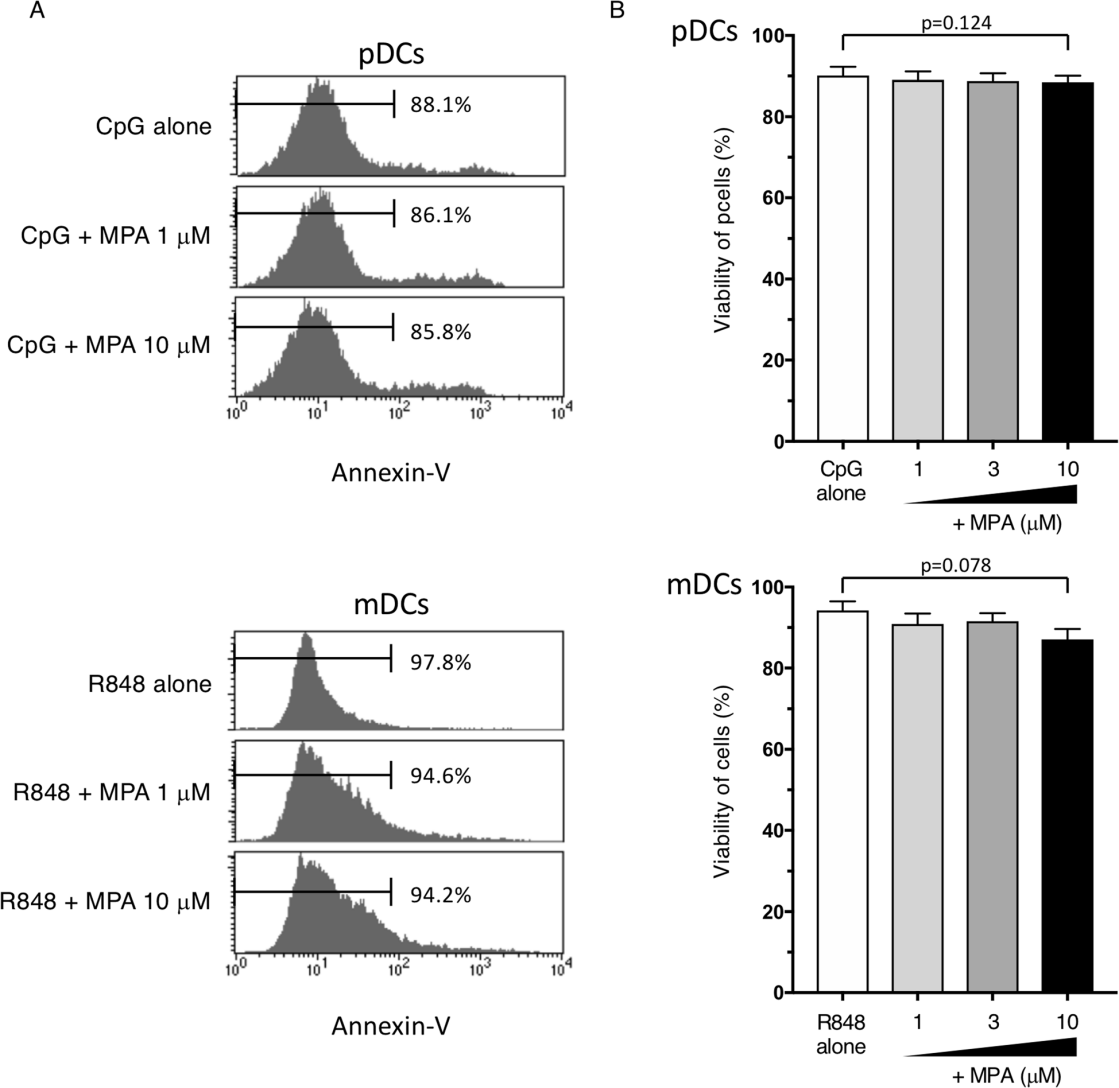
Effects of MPA on the survival of human DC subsets. Purified human pDCs and mDCs were incubated for 24 h with different concentrations of MPA (1−10 μM) or vehicle, in the presence of CpG-ODN and R848, respectively. Viable cells were measured by annexin-V staining (**a**, **b**). The annexin-V staining profile of one representative experiment out of five independent experiments is shown (**a**). Percentages of annexin V-negative cells are shown in **a** and **b**. One set of experiments was performed on DCs from one donor, and the data are shown as the mean ± SEM of five independent donors. Statistical significance was determined using a paired *t* test

**Fig. 2:**
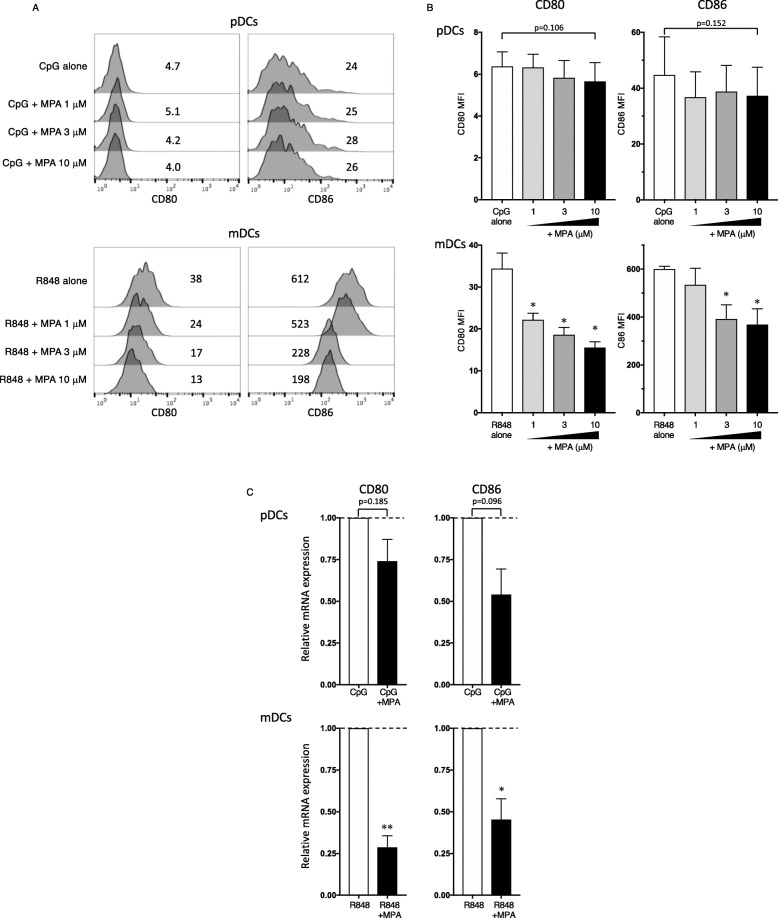
Effects of MPA on the maturation of human DC subsets. **a**, **b** Purified human pDCs and mDCs were incubated for 24 h with different concentrations of MPA (1−10 μM) or vehicle, in the presence of CpG-ODN and R848, respectively. The expression of CD80 and CD86 was analyzed by flow cytometry. The staining profiles of CD80 and CD86 of one representative experiment out of at least four independent experiments are shown (**a**). The mean fluorescence intensity (MFI) of CD80 and CD86 is shown (**a**, **b**). One set of experiments was performed on DCs from one donor, and the data are shown as the mean ± SEM of four (pDCs) or five (mDCs) independent donors. **c** After 8 h of culture with 10 μM MPA or vehicle, CD80 and CD86 mRNA in the DC subsets was analyzed by real-time PCR. One set of experiments was performed on DCs from one donor, and the data are shown as the mean ± SEM of three independent donors. Statistical significance was determined using a paired *t* test (*p < 0.05, **p < 0.01)

Clinical pharmacokinetics show that the mean clinical trough plasma MPA concentration of lupus nephritis female patients who were administered 1500 mg twice a day of oral MMF is 10.6 μM (3.4 mg/L) [23]. Another clinical study reported that the plasma concentration of MPA at trough in patients with severe lupus nephritis is 7.7 μM (2.47 mg/L) [24]. Therefore, we used 1−10 μM MPA (covering the clinical trough plasma concentration range for MMF/MPA) in our experiments. Concentrations of up to 10 μM of MPA did not induce apoptosis of pDCs or mDCs in response to CpG-ODN or R848, respectively (Fig. 1a and b). The clinical trough concentration of MPA downregulated the R848-induced surface expression levels of CD80 and CD86 (Fig. 2a and b) and mRNA levels (Fig. 2c) in mDCs. By contrast, pDCs stimulated with CpG-ODN did not substantially upregulate either CD80 or CD86 expression (Fig. 2a), and the addition of MPA slightly but not significantly decreased these expression levels (Fig. 2b and c).

### MPA inhibits IFN-α production from human pDCs

**Fig. 3:**
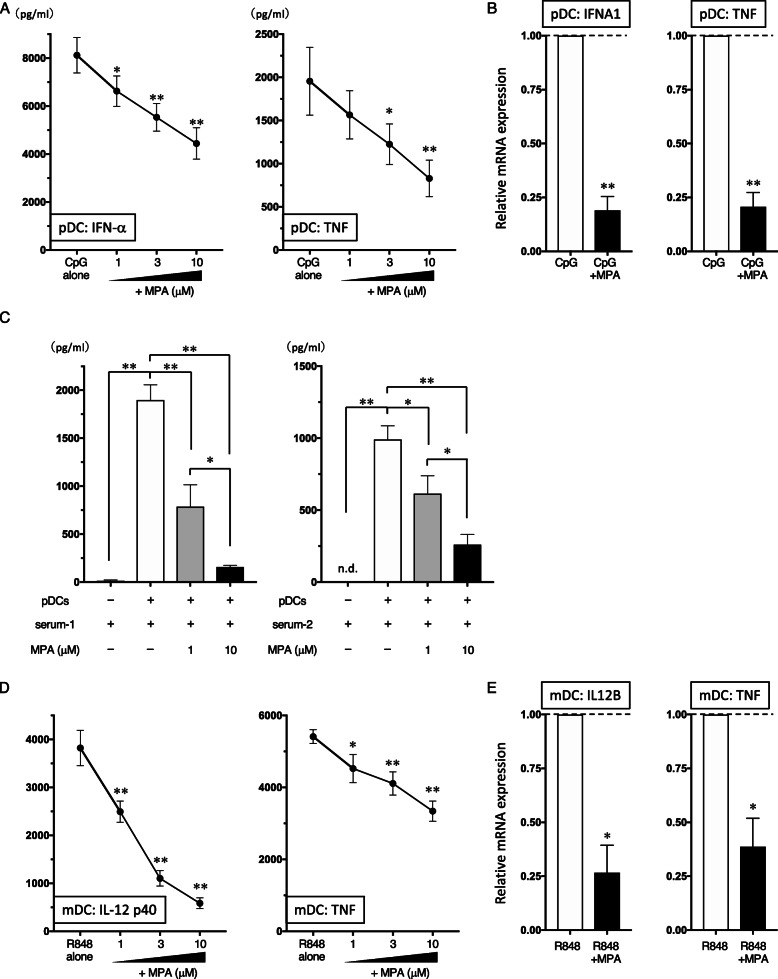
Effects of MPA on the ability of human DC subsets to produce cytokines. **a**, **d** Purified human pDCs and mDCs were incubated with different concentrations of MPA (1−10 μM) or vehicle, in the presence of CpG-ODN and R848, respectively. After 24 h, the concentrations of the indicated cytokines in the culture supernatants of pDCs (**a**) and mDCs (**d**) were measured by ELISA. One set of experiments was performed on DCs from one donor, and the data are shown as the mean ± SEM of eight (for pDC-IFN-α), four (for pDC-TNF), five (for mDC-IL-12), or six (for mDC-TNF) independent donors. **b**, **e** After 8 h of culture with 10 μM MPA or vehicle, the mRNA levels of IFNA1, IL12B, and TNF were analyzed in the DC subsets by real-time PCR. One set of experiments was performed on DCs from one donor, and the data are shown as the mean ± SEM of three independent donors. **c** pDCs were incubated with MPA (1 or 10 μM) or vehicle in serum-free RPMI, and serum (serum-1 or serum-2) from two SLE patients (at a final concentration of 20% vol/vol) was added. After 24 h, the concentrations of IFN-α in the culture supernatants were measured by ELISA. One set of experiments was performed on pDCs from one donor, and the data are shown as the mean ± SEM of five independent donors. Statistical significance was determined using a paired *t* test (*p < 0.05, **p < 0.01). n.d., not detected

The secretion of type I IFN is a key molecular event that initiates and promotes the autoimmune process [15], and pDCs are the major source of type I IFN in PBMCs [9]. Thus, we next examined whether MPA could inhibit type I IFN production by pDCs. Notably, MPA dose-dependently inhibited the production of both IFN-α and TNF by pDCs in response to stimulation with CpG-ODN (Fig. 3a), and this was confirmed by transcription levels (Fig. 3b).

Immune complexes in serum consisting of autoantibodies and self-DNA are a pathogenic trigger for the induction of aberrant production of type I IFN by blood pDCs, which drives autoimmunity in SLE [15]. SLE serum also contains a nuclear protein (high-mobility group box 1 protein [HMGB1]) or an antimicrobial peptide LL37 released from damaged tissues or neutrophils [15, 16, 25]; these host-derived transporter agents are required for triggering the SLE pathogenic process. Thus, SLE sera possess a complete set of essential components for inducing pathogenic pDC-derived type I IFN. In previous in vitro experiments, stimulation of PBMCs with serum obtained from a patient with SLE induced IFN-α production [22, 26, 27]. Accordingly, we next investigated whether MPA could inhibit SLE serum-induced IFN-α production under conditions mimicking the pathophysiological conditions of SLE. We preliminarily tested sera from five patients with active SLE who had anti-double-stranded DNA antibody, from which we selected the two best sera for inducing IFN-α in healthy PBMCs (data not shown). SLE serum (either serum-1 or serum-2) induced a significant level of IFN-α from pDCs (Fig. 3c). Notably, concentrations of MPA of up to 10 μM significantly repressed SLE serum-induced IFN-α production in a dose-dependent manner (Fig. 3c). These data suggest that MPA has a potential inhibitory effect on the IFN-α production triggered by SLE pathogenic conditions.

### MPA interferes with the nuclear translocation of IRF7 in pDCs

**Fig. 4:**
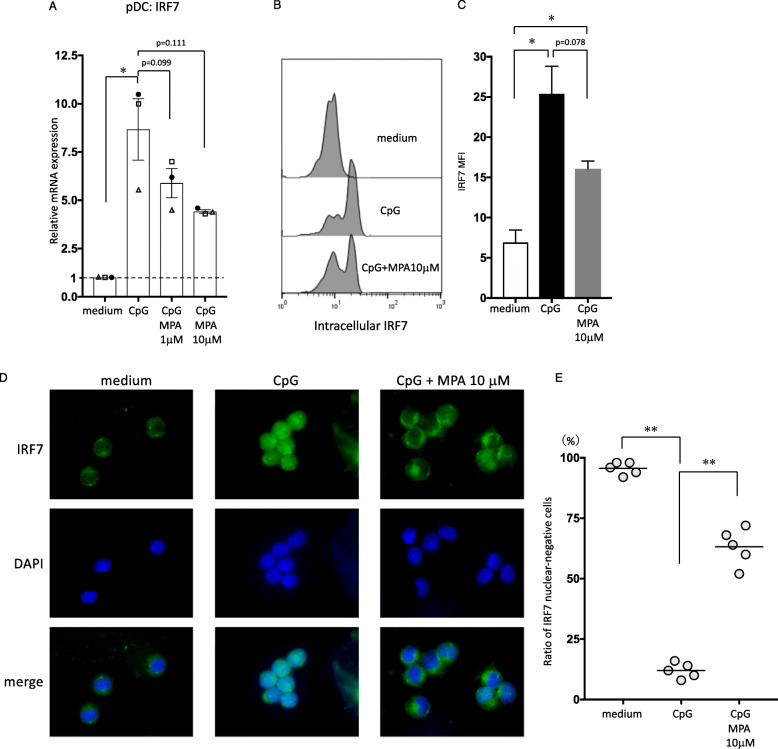
Effect of MPA on the nuclear translocation of IRF7 in pDCs. Purified human pDCs were incubated with MPA (1 or 10 μM) or vehicle, followed by the addition of medium alone or CpG-ODN. **a** After 4 h of culture, IRF7 mRNA in the DC subsets was analyzed by real-time PCR. One set of experiments was performed on DCs from one donor, and the data are shown as the mean ± SEM of three independent donors. **b**, **c** After 3 h of culture, the expression of intracellular IRF7 was analyzed by flow cytometry. The staining profile of IRF7 for one representative experiment out of three independent experiments is shown (**b**). The mean fluorescence intensity (MFI) of IRF7 is shown, and one set of experiments was performed on pDCs from one donor, and the data are shown as the mean ± SEM of three independent donors (**c**). **d** After 3 h of culture, pDCs were visualized by immunofluorescence with anti-IRF7 antibody (green) and nuclei staining with DAPI (blue). Similar results were observed in all five independent donors, and representative images are shown. **e** For measuring the IRF-7 translocation in cell nuclei, stained cells on each slide were counted. Cells were regarded as negative when the IRF7 expression level in the cytoplasm was higher and distinguishable from that in the nucleus. The ratio of nuclear IRF7-negative cells was analyzed based on 50 cells from each donor. One set of experiments was performed on pDCs from one donor, and the data are shown as the mean ± SEM of five independent donors. Statistical significance was determined using a paired *t* test (*p < 0.05, **p < 0.01)

Because the essential molecular step in type I IFN production by pDCs has been shown to be the nuclear translocation of constitutively expressed IRF7 [21, 28], we next examined whether MPA alters this process by evaluating both the quantitative transcriptional level of IRF-7 and the migration of IRF7 into the nucleus upon activation. First, we performed real-time PCR assays, and the results showed that the level of IRF-7 RNA in pDCs was significantly upregulated following CpG stimulation (Fig. 4a). Although the transcriptional upregulation of IRF-7 trended to lower following treatment with MPA, this difference did not reach statistical significance. The analysis of intracellular IRF7 expression showed similar results, with lower fluorescence intensity of IRF7 being observed in pDCs following treatment with MPA (Fig. 4b and c). Analysis with immunofluorescence microscopy revealed that IRF7 was localized in the cytoplasmic area of unstimulated pDCs (Fig. 4d). After stimulation with CpG-ODN, IRF7 expression was detected in the nucleus, as shown by its colocalization with DAPI nuclear staining, indicating the nuclear translocation of IRF7. This nuclear staining of IRF7 and the colocalization of IRF7 and DAPI staining induced by CpG stimulation was prevented by the presence of 10 μM MPA (Fig. 4d). Thus, MPA helped retain IRF7 in the cytoplasm, indicating its inhibitory effect on IRF7 nuclear translocation in pDCs. To quantify this finding, we counted the numbers of pDCs on the slide with or without nuclear IRF7 expression, as described in the “Material and methods” section and in a previous study [10]. Almost all unstimulated pDCs were found to be nuclear IRF7-negative (mean ± SEM: 95.6% ± 1.2%), and CpG stimulation significantly reduced the frequency of cells without IRF7 nuclear translocation (12.0% ± 1.4%) (Fig. 4e). Treatment with MPA significantly augmented the frequency of nuclear IRF7-negative cells following stimulation with CpG (63.2% ± 3.4%) (Fig. 4e). Thus, our result indicates that MPA acts as an inhibitor of IRF7 nuclear translocation.

**Fig. 5:**
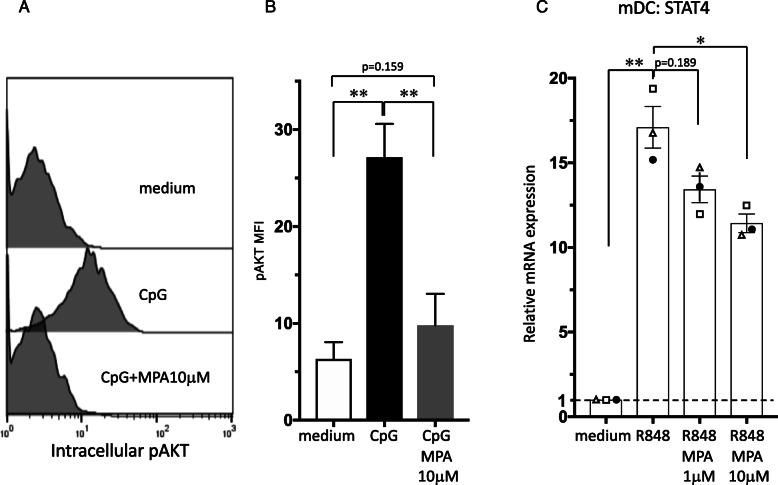
Effect of MPA on intracellular molecules in DC subsets. Purified human pDCs and mDCs were incubated with MPA (1 or 10 μM) or vehicle, followed by the addition of medium alone, CpG-ODN, or R848. **a**, **b** After 90 min of culture, the expression of intracellular pAKT was analyzed by flow cytometry. The staining profile of pAKT for one representative experiment out of three independent experiments is shown (**a**). The MFI of pAKT is shown and one set of experiments was performed on pDCs from one donor, and the data are shown as the mean ± SEM of three independent donors (**b**). **c** After 4 h of culture, STAT4 in the mDC subsets was analyzed by real-time PCR. One set of experiments was performed on DCs from one donor, and the data are shown as the mean ± SEM of three independent donors. Statistical significance was determined using a paired *t* test (*p < 0.05, **p < 0.01)

To provide additional insight into possible mechanisms through which MMF is mediating the actions inhibiting the nuclear translocation of IRF7, we focused on phosphorylated AKT (pAKT) as a downstream target of phosphoinositide 3-kinase (PI3K). PI3K-pAKT pathway is reported to be closely involved in nuclear translocation of IRF7 in TLR-dependent type I IFN production of pDCs [29]. Intracellular pAKT expression in pDCs was upregulated by CpG-ODN, and this increase was repressed by the addition of MPA treatment (Fig. 5a and b).

### MPA inhibits Th1-related responses in mDCs

In the SLE pathogenic spiral, activated mDCs play a role in the induction of autoreactive effectors such as CD4^+^ T cells, CD8^+^ T cells, and B cells. When stimulated with TLR ligands, mDCs are able to induce a Th1 response. To investigate the immunomodulatory actions of MPA on the function of human mDCs in Th1-related activation pathways, we analyzed the cytokine production by mDCs that had been stimulated with R848. MPA inhibited the R848-induced production of IL-12 p40 and TNF in a dose-dependent manner (Fig. 3d) and this inhibitory activity was also observed with mRNA levels (Fig. 3e). On the basis of the results of real-time PCR on mDCs, R848 significantly upregulated STAT4 mRNA, which is important for DC-mediated Th1 responses and IL-12 production [30, 31]. Notably, we found that 10 μM MPA significantly downregulated R848-induced STAT4 mRNA (Fig. 5c).

## Discussion

In NZB mice, which develop an immune complex disease with nephritis that is similar to human SLE, MMF has a beneficial effect on both renal and overall survival [32]. MMF has been introduced as a novel immunosuppressive agent in SLE, often in patients who are intolerant or resistant to conventional immunosuppressive regimens [33, 34]. Although outlined responses are described in prior publications, the mechanism of action of MMF/MPA in the SLE pathogenic spiral remains poorly understood, except for the direct suppressive effects on T and B lymphocytes. In patients with active SLE, auto-anti-DNA antibody, such as flares of lupus glomerulonephritis, is a common and significant marker [35–38]. Sustained activation of pDCs in the blood by immune complexes induces dysregulated type I IFN elevation, as a central molecular event, which subsequently causes mDC activation and the resultant induction of autoreactive lymphocytes in the vicious autoimmune spiral of SLE [15, 16]. Here, we focused on the function of human DC subsets involved in this pathogenic spiral and unveiled the inhibitory potential of MPA against such pDC-derived IFN-α production and mDC function. We found that MPA exerted an inhibitory action on IRF7 nuclear translocation in pDCs. It has been reported that the PI3K-AKT pathway is critically involved in the nuclear translocation of IRF7 in pDCs [29]. Our data suggest that MPA may inhibit nuclear translocation of IRF7 possibly through suppressing AKT activity in pDCs. On the basis of our results, treatment with MPA may be able to interrupt the autoimmune pathogenic spiral by limiting the disordered production of type I IFN that occurs under the pathophysiological conditions of SLE. Thus, the present study revealed a possible mechanism underlying the therapeutic potential of MMF/MPA for treating SLE by targeting pDCs. Notably, trough plasma concentrations of MPA did not induce cell apoptosis of either pDCs or mDCs, suggesting that MPA at clinical doses does not cause excessive immunosuppression. Hence, MMF may be useful as a long-term maintenance therapy for SLE.

Hydroxychloroquine is an effective therapeutic agent for SLE and has a mechanism similar to that of MPA; it is a strong TLR-signaling inhibitor that suppresses the production of type I IFN by pDCs [39]. MMF/MPA may exhibit cellular effects via mechanisms of action similar to those by which hydroxychloroquine treats autoimmunity [40]. Accumulating evidence has substantiated the importance of TLRs in the pathogenesis and progression of autoimmune processes in SLE and its renal components [41]. On the basis of the common properties of these two drugs, inhibition of the pDC-IFN axis may be an effective SLE treatment strategy. In fact, inhibition of TLR9 signaling with synthetic ODN can also exhibit beneficial effects on the progression of lupus nephritis in MRL^lpr/lpr^ mice [42].

The present study also revealed the ability of MPA, used at the clinical trough level, to impair the maturation and Th1-inducing capacity of DCs by downregulating STAT4. In support of our findings, a recent in vitro study showed that 20 μM MMF can inhibit the LPS-induced expression of MHC-class II, CD86, CD40, CD83, and NF-κB in human blood mDCs [20]. Moreover, MMF was reported to suppress the differentiation of human monocyte-derived DCs [18, 19]. These immunosuppressive effects of MMF/MPA on mDCs may further contribute to its ability to break the pathogenic spiral of SLE.

Ideally, pDCs from the blood of SLE patients would have been used for evaluating the ability of MPA to suppress type I IFN production, triggered by pathogenic conditions. However, the blood of SLE patients has greatly reduced the numbers of circulating pDCs [16]. Furthermore, the numbers of pDCs in the bone marrow are very low in lupus-prone mice [43]. Therefore, because it was logistically and ethically difficult to perform these experiments using pDCs from SLE patients, we designed an experimental system using SLE sera.

## Conclusions

To the best of our knowledge, this is the first study to investigate the effect of MMF/MPA on pDCs. This work focusing on DC function clarified one of the possible mechanisms by which MPA could be clinically effective in the treatment of SLE. Our findings suggest that MMF may be able to interrupt the vicious pathogenic spiral of autoimmune disorders, not only by inhibiting T and B lymphocytes but also by regulating the function of DC subsets.

## Supplementary information

**Additional file 1: Supplementary Figure 1.** Isolation of blood DC subsets. Blood pDCs and mDCs were detected and isolated as the CD11c^−^BDCA-4^+^ population and CD11c^+^BDCA-4^−^ population, respectively, in the fraction of DC-enriched PBMCs (lineage [CD3, CD14, CD15, CD16, CD19, and CD56]-negative and CD4-positive) after immunobead-selection (CD3- and CD14-beads negative selection and subsequent CD4-bead positive selection) from total PBMCs. A representative flow cytometry analysis performed to detect pDCs and mDCs in PBMCs from healthy donors is shown. The numbers indicate the percentages within the gated fractions.

## Data Availability

The data that support the findings of this study are available on request from the corresponding author (T.I.). The data are not publicly available owing to information that could compromise research participant privacy.

## Abbreviations

MMF: Mycophenolate mofetil
MPA: Mycophenolic acid
BDCA: Blood dendritic cell antigen
DC: Dendritic cells
IFN: Interferon
IKK: IκB kinase
IRF7: IFN regulatory factor 7
PBMC: Peripheral blood mononuclear cells
pDC: Plasmacytoid dendritic cell
SLE: Systemic lupus erythematosus
TNF: Tumor necrosis factor
TLR: Toll-like receptor
