# Supplementary figures and images for: Mycophenolic acid, the active form of mycophenolate mofetil, interferes with IRF7 nuclear translocation and type I IFN production by plasmacytoid dendritic cells
Source: Arthritis Res Ther. 2020 Nov 9;22:264. doi: 10.1186/s13075-020-02356-z (PMC7654586; doi:10.1186/s13075-020-02356-z)

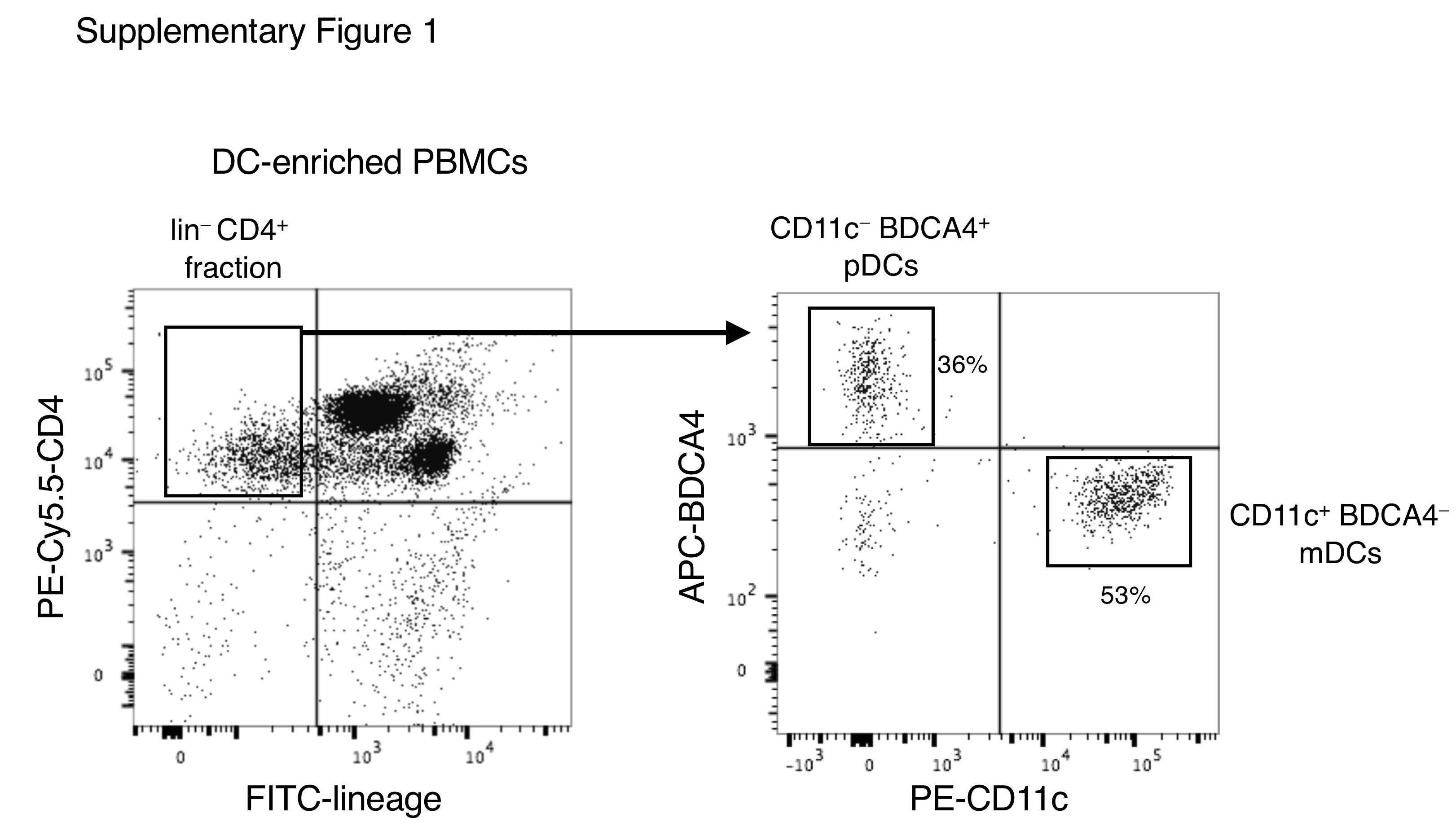

Supplement: Supplementary file 1 — Additional file 1: Supplementary Figure 1. Isolation of blood DC subsets. Blood pDCs and mDCs were detected and isolated as the CD11c−BDCA-4+ population and CD11c+BDCA-4− population, respectively, in the fraction of DC-enriched PBMCs (lineage [CD3, CD14, CD15, CD16, CD19, and CD56]-negative and CD4-positive) after immunobead-selection (CD3- and CD14-beads negative selection and subsequent CD4-bead positive selection) from total PBMCs. A representative flow cytometry analysis performed to detect pDCs and mDCs in PBMCs from healthy donors is shown. The numbers indicate the percentages within the gated fractions. [file 13075_2020_2356_MOESM1_ESM.png]
